# Supplementary material for: Comparative epidemic expansion of SARS-CoV-2 variants Delta and Omicron in the Brazilian State of Amazonas
Source: Nat Commun. 2023 Apr 11;14:2048. doi: 10.1038/s41467-023-37541-6 (PMC10089528; doi:10.1038/s41467-023-37541-6)
Supplement: Supplementary file 1 — Supplementary Information [file 41467_2023_37541_MOESM1_ESM.pdf]

SUPPLEMENTARY TABLES

Table S1: Pango lineages detected in Amazonas state.

Lineage		N	N (%)	Lineage		N	N (%)
	AY.4	1	<1		BA.1	1,132	53
	AY.6	1	<1		BA.1.1	72	3
	AY.9.2	105	11		BA.1.1.10	1	<1
	AY.20	4	<1		BA.1.1.14	11	1
	AY.34	19	2		BA.1.1.15	204	10
	AY.34.1	2	<1		BA.1.1.18	2	<1
	AY.34.1.1	4	<1		BA.1.9	115	5
	AY.34.2	1	<1		BA.1.14.1	82	4
	AY.42	5	1	**Omicron**	BA.1.14.2	37	2
	AY.43	107	11		BA.1.14	19	1
	AY.43.1	1	<1		BA.1.15	48	2
**Delta**	AY.59	1	<1		BA.1.16	1	<1
	AY.99.1	1	<1		BA.1.17	4	<1
	AY.99.2	496	51		BA.1.17.2	355	17
	AY.100	3	<1		BA.1.18	1	<1
	AY.101	57	6		BA.1.20	51	2
	AY.103	2	<1		**Total**	**2,135**	**100**
	AY.116	1	<1				
	AY.119	2	<1				
	AY.119.1	3	<1				
	AY.122	147	15				
	B.1.617.2	2	<1				
	**Total**	**965**	**100**				

The table details the absolute and relative frequencies of the Pango lineages to which sequences generated in Amazonas state between July-2021 and March-2022 were assigned. Only sequences in the VOCs Delta and Omicron were listed.

Table S2: Major clades of the VOC Delta detected in Amazonas state.

Lineage	Cluster	N (%)	Entry Point (PSP)	Source (PSP)
AY.9.2	AY.9.2$_{\text{AM-I}}$	88 (84)	Manaus (74)	SA North (91)
AY.43	AY.43$_{\text{AM-I}}$	32 (30)	Tefé (100)	EU (99)
	AY.43$_{\text{AM-II}}$	30 (28)	Manaus (100)	SA North (45)
AY.99.2	AY.99.2$_{\text{AM-I}}$	95 (19)	Manaus (100)	BR-SE (92)
	AY.99.2$_{\text{AM-II}}$	85 (17)	Manaus (95)	BR-SE (96)
	AY.99.2$_{\text{AM-III}}$	50 (10)	Manaus (99)	BR-CW (100)
	AY.99.2$_{\text{AM-IV}}$	40 (8)	Manaus (98)	BR-SE (100)
	AY.99.2$_{\text{AM-V}}$	24 (5)	Parintins (51)	BR-N (70)
	AY.99.2$_{\text{AM-VI}}$	23 (5)	Manicoré (69)	BR-N (97)
	AY.99.2$_{\text{AM-VII}}$	22 (4)	Manaus (98)	BR-N (99)
	AY.99.2$_{\text{AM-VIII}}$	19 (4)	Parintins (99)	BR-N (86)
	AY.99.2$_{\text{AM-IX}}$	16 (3)	Parintins (99)	BR-N (76)
AY.101	AY.101$_{\text{AM-II}}$	45 (79)	Manaus (97)	BR-S (99)
AY.122	AY.122$_{\text{AM-I}}$	100 (68)	Manaus (97)	SA North (88)
	AY.122$_{\text{AM-II}}$	17 (12)	Manaus (98)	SA North (62)

The table details the major clusters detected in Amazonas state of the VOC Delta, defined by their statistical support (aLRT > 0.80), location of their MRCA (PSP of Amazonas location > 0.80), and dimension (at least 1% of all sequences from the VOC). For each cluster are supplied absolute and relative dimensions, the most probable entry point among Amazonas's regions, and the most probable origin of the migration it originated from, the last two accompanied by their PSP values. BR: Brazil, EU: Europe, SA: South America, N: north, S: south, SE: southeast, CW: central-west.

Table S3: Major clades of the VOC Omicron detected in Amazonas state.

Lineage	Cluster	N (%)	Entry Point (PSP)	Source (PSP)
BA.1	BA.1$_{AM-I}$	185 (16)	Manaus (100)	BR-SE (78)
	BA.1$_{AM-II}$	168 (15)	Manaus (99)	BR-SE (78)
	BA.1$_{AM-III}$	55 (5)	Manaus (100)	BR-SE (78)
	BA.1$_{AM-IV}$	49 (4)	Parintins (100)	BR-SE (95)
	BA.1$_{AM-V}$	38 (3)	Manaus (99)	EU (99)
	BA.1$_{AM-VI}$	37 (3)	Manaus (85)	EU (100)
	BA.1$_{AM-VII}$	29 (3)	Manaus (100)	BR-SE (78)
	BA.1$_{AM-VIII}$	27 (2)	Manaus (96)	BR-SE (81)
	BA.1$_{AM-IX}$	27 (2)	Tefé (100)	BR-SE (83)
	BA.1$_{AM-X}$	24 (2)	Manaus (100)	BR-SE (78)
BA.1.1.15	BA.1.1.15$_{AM-I}$	165 (81)	Manaus (100)	EU (100)
	BA.1.1.15$_{AM-II}$	26 (13)	Manaus (100)	EU (100)
BA.1.9	BA.1.9$_{AM-I}$	35 (30)	Manaus (100)	BR-SE (70)
BA.1.14.1	BA.1.14.1$_{AM-I}$	21 (26)	Manaus (89)	BR-SE (80)
BA.17.2	BA.17.2$_{AM-I}$	50 (14)	Manaus (100)	EU (83)
	BA.17.2$_{AM-II}$	20 (6)	Manaus (99)	EU (83)
BA.1.20	BA.1.20$_{AM-I}$	50 (98)	Manaus (100)	BR-SE (97)

The table details the major clusters detected in Amazonas state of the VOC Omicron, defined by their statistical support (aLRT > 0.80), location of their MRCA (PSP of Amazonas location > 0.80), and dimension (at least 1% of all sequences from the VOC). For each cluster are supplied absolute and relative dimensions, the most probable entry point among Amazonas's regions, and the most probable origin of the migration it originated from, the last two accompanied by their PSP values. BR: Brazil, EU: Europe, N: north, SE: southeast.

SUPPLEMENTARY FIGURES:

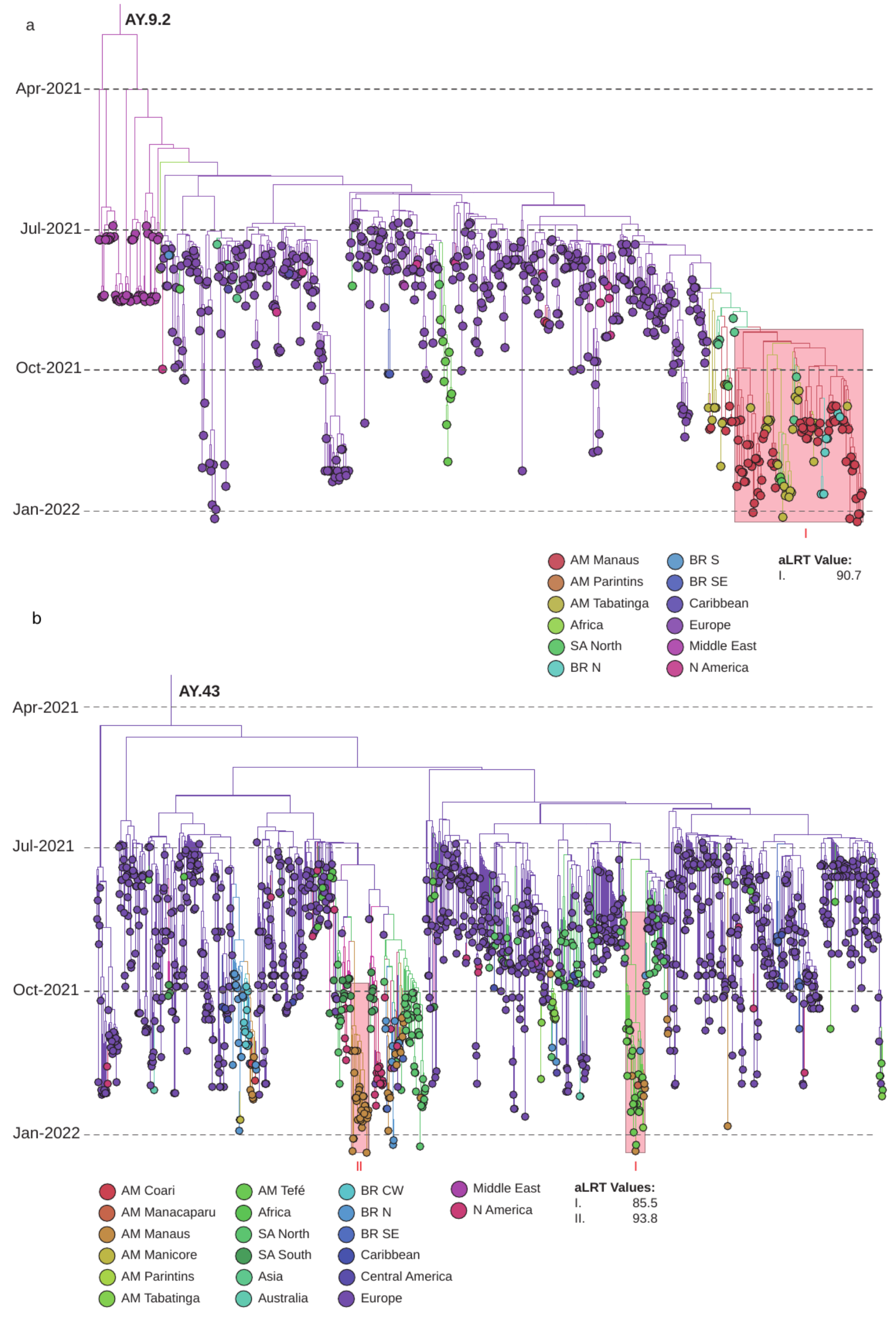

Figure S1: Spatiotemporal characterization of lineages AY.9.2 and AY.43 in Amazonas state. Time-scaled MCC tree of SARS-Cov-2 lineages AY.9.2 (n_{TOTAL} = 616) and AY.43 (n_{TOTAL}= 1,339). Branches are colored according to their most probable location, based on the color scheme shown in the legends. Large clusters identified in each lineage are indicated, as are their aLRT support obtained in the previous ML analysis. AM: Amazonas, N: north, S: south, SA: South America, SE: southeast, CW: central-west.

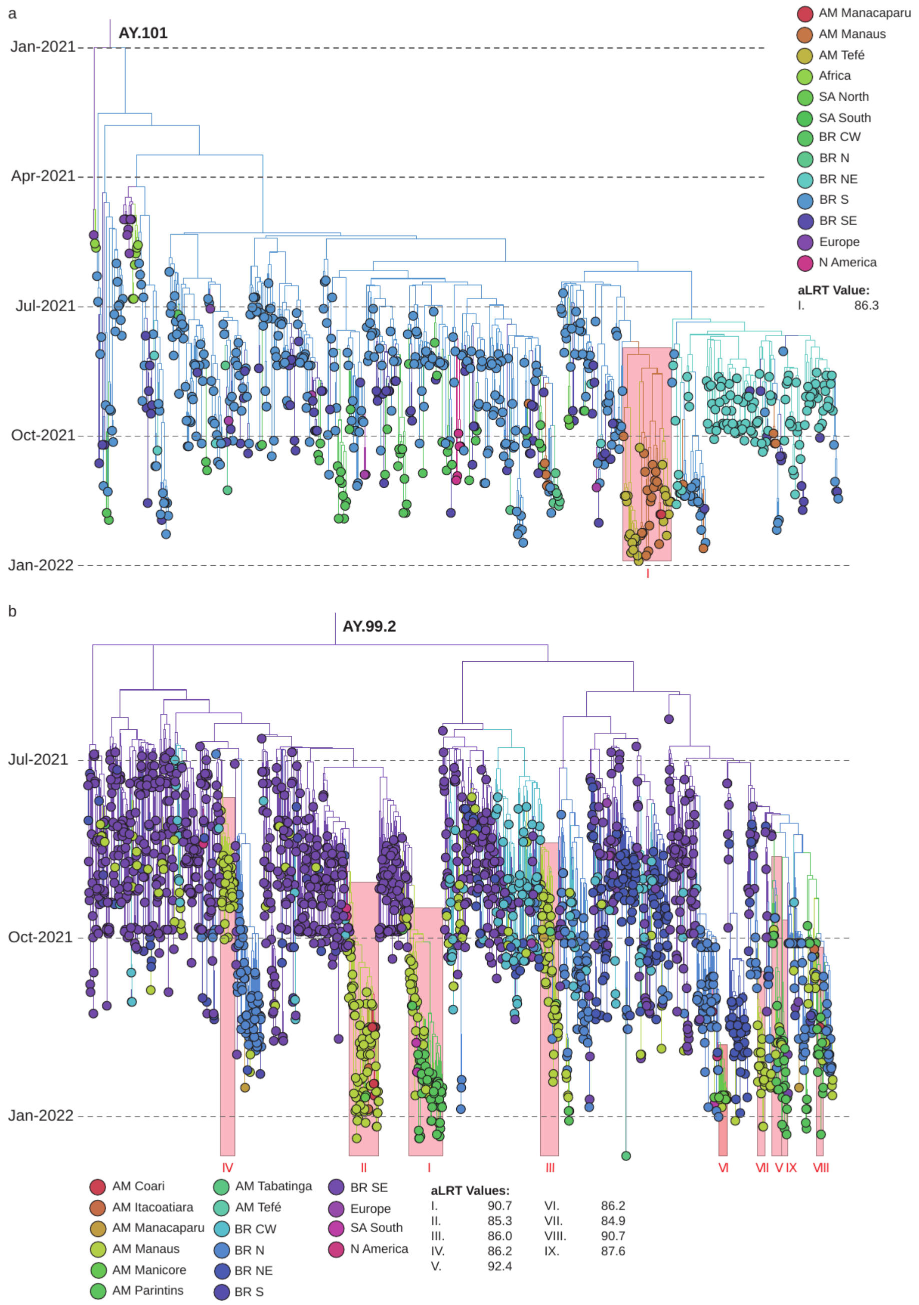

Figure S2: Spatiotemporal characterization of lineages AY.101 and AY.99.2 in Amazonas state. Time-scaled MCC tree of SARS-Cov-2 lineages AY.101 (n_{TOTAL} = 691) and AY.99.2 (n_{TOTAL}= 2,102). Branches are colored according to their most probable location, based on the color scheme shown in the legends. Large clusters identified in each lineage are indicated, as are their aLRT support obtained in the previous ML analysis. AM: Amazonas, N: north, S: south, SA: South America, SE: southeast, CW: central-west.

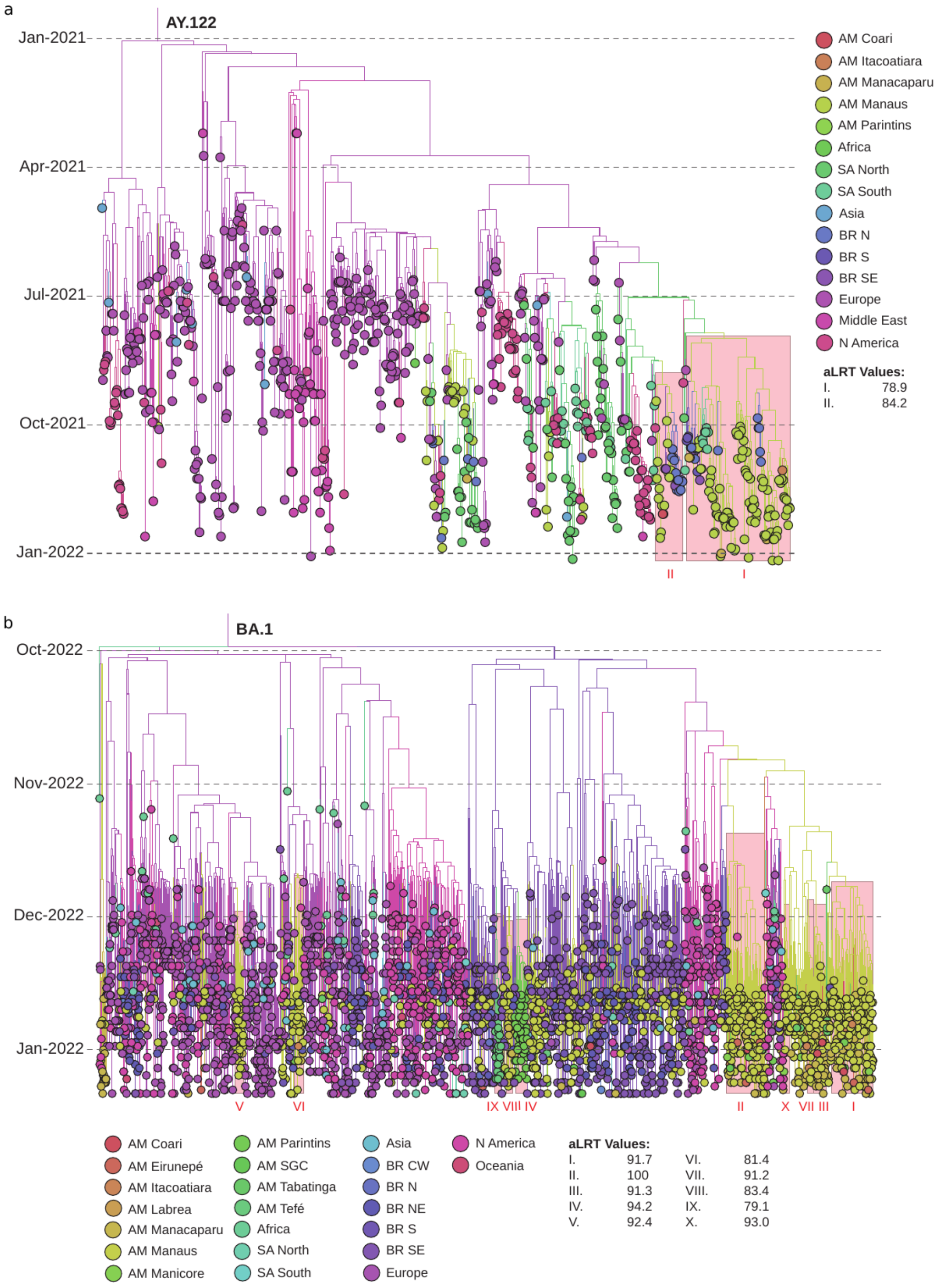

Figure S3: Spatiotemporal characterization of lineages AY.122 and BA.1 in Amazonas state. Time-scaled MCC tree of SARS-Cov-2 lineages AY.122 (n_{TOTAL} = 839) and BA.1 (n_{TOTAL}= 3,445). Branches are colored according to their most probable location, based on the color scheme shown in the legends. Large clusters in each lineage are indicated alongside the aLRT support obtained in the previous ML analysis. AM: Amazonas, N: north, NE: northeast, S: south, SA: South America, SE: southeast, CW: central-west.

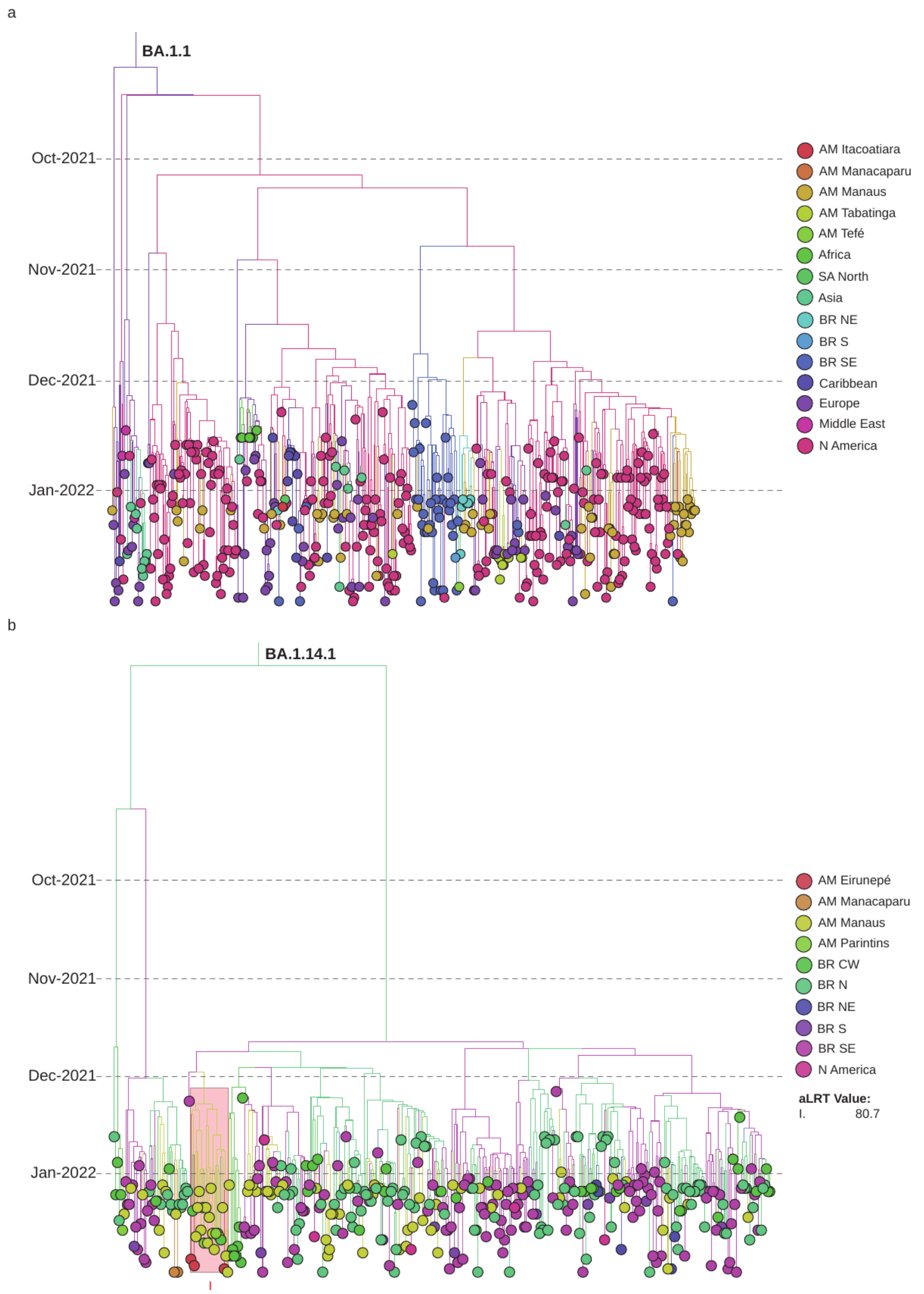

Figure S4: Spatiotemporal characterization of lineages BA.1.1 and BA.1.14.1 in Amazonas state. Time-scaled MCC tree of SARS-Cov-2 lineages BA.1.1 (n_{TOTAL} = 478) and BA.1.14.1 (n_{TOTAL}= 394). Branches are colored according to their most probable location, based on the color scheme shown in the legends. Large clusters in each lineage are indicated alongside the aLRT support obtained in the previous ML analysis. AM: Amazonas, N: north, NE: northeast, S: south, SA: South America, SE: southeast, CW: central-west.

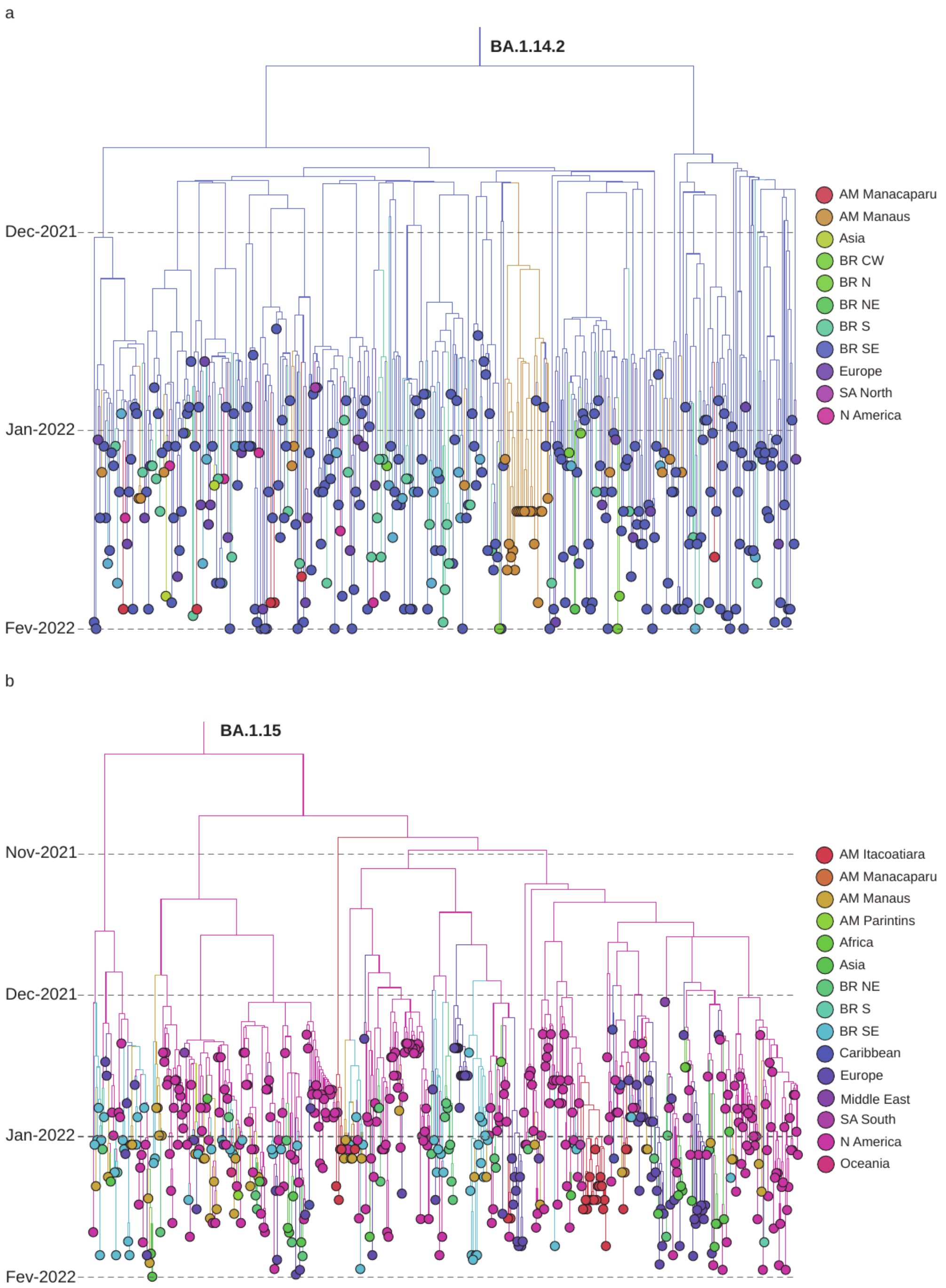

Figure S5: Spatiotemporal characterization of lineages BA.1.14.2 and BA.1.15 in Amazonas State. Time-scaled MCC tree of SARS-Cov-2 lineages BA.1.14.2 (n_{TOTAL} = 363) and BA.1.15 (n_{TOTAL}= 526). Branches are colored according to their most probable location, based on the color scheme shown in the legends. AM: Amazonas, N: north, NE: northeast, S: south, SA: South America, SE: southeast, CW: central-west.

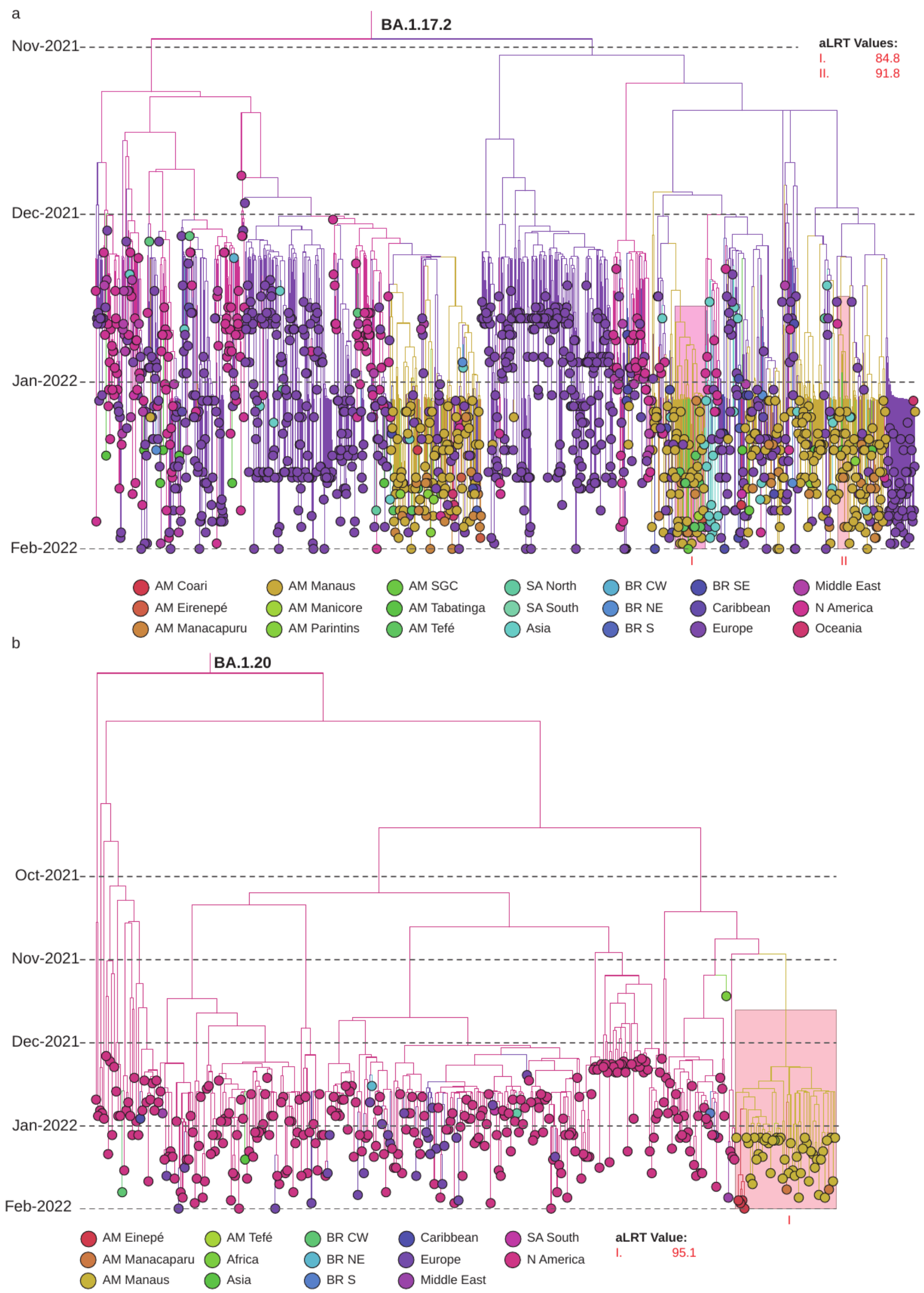

Figure S6: Spatiotemporal characterization of lineages BA.1.1 and BA.1.14.1 in Amazonas state. Time-scaled MCC tree of SARS-Cov-2 lineages BA.1.1 (n_{TOTAL} = 478) and BA.1.14.1 (n_{TOTAL}= 394). Branches are colored according to their most probable location, based on the color scheme shown in the legends. Large clusters in each lineage are indicated alongside the aLRT support obtained in the previous ML analysis. AM: Amazonas, N: north, NE: northeast, S: south, SA: South America, SE: southeast, CW: central-west.

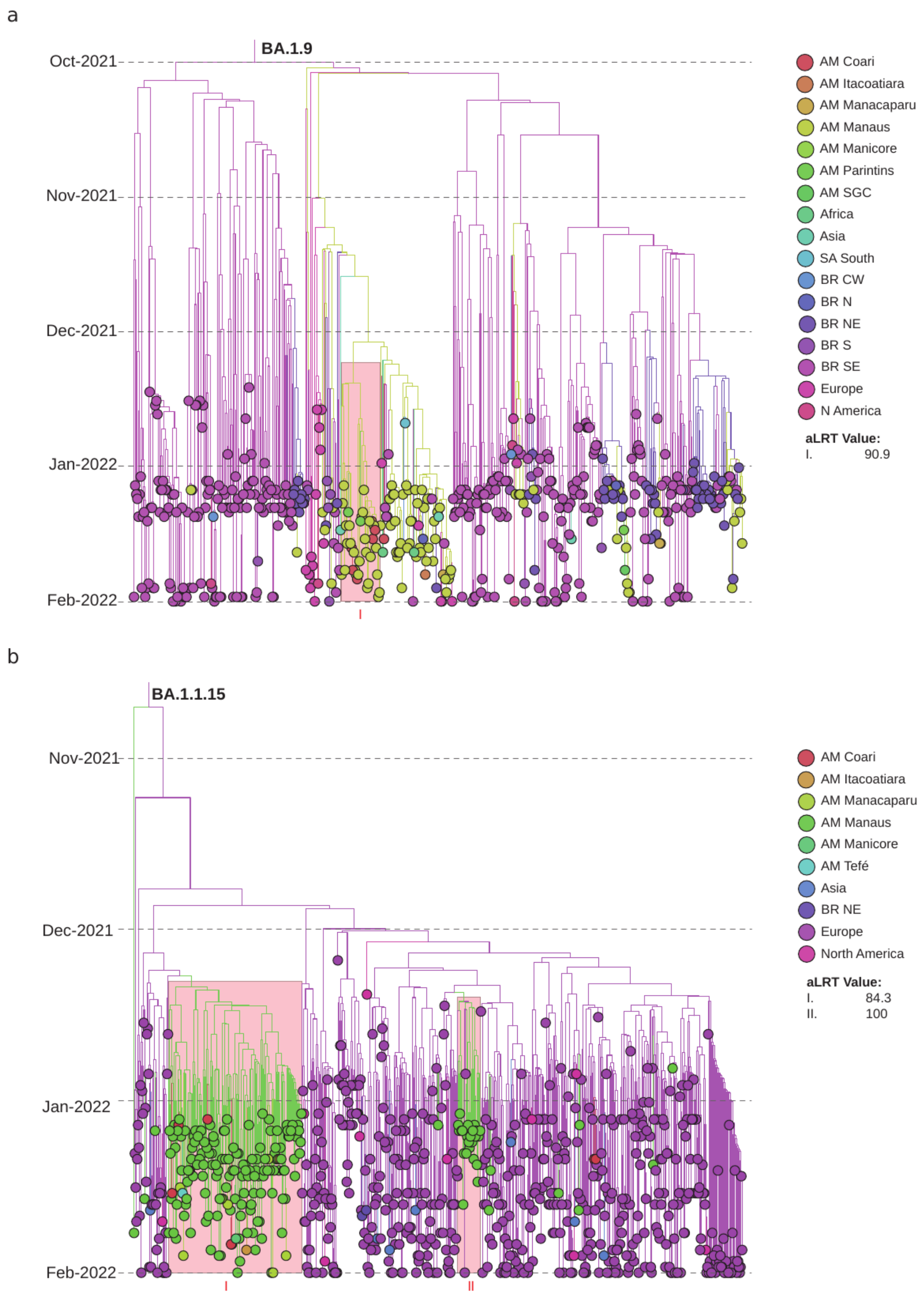

Figure S7: Spatiotemporal characterization of lineages BA.1.9 and BA.1.1.15 in Amazonas state. Time-scaled MCC tree of SARS-Cov-2 lineages BA.1.1 (n_{TOTAL} = 478) and BA.1.14.1 (n_{TOTAL}= 394). Branches are colored according to their most probable location, based on the color scheme shown in the legends. Large clusters in each lineage are indicated alongside the aLRT support obtained in the previous ML analysis. AM: Amazonas, N: north, NE: northeast, S: south, SA: South America, SE: southeast, CW: central-west.

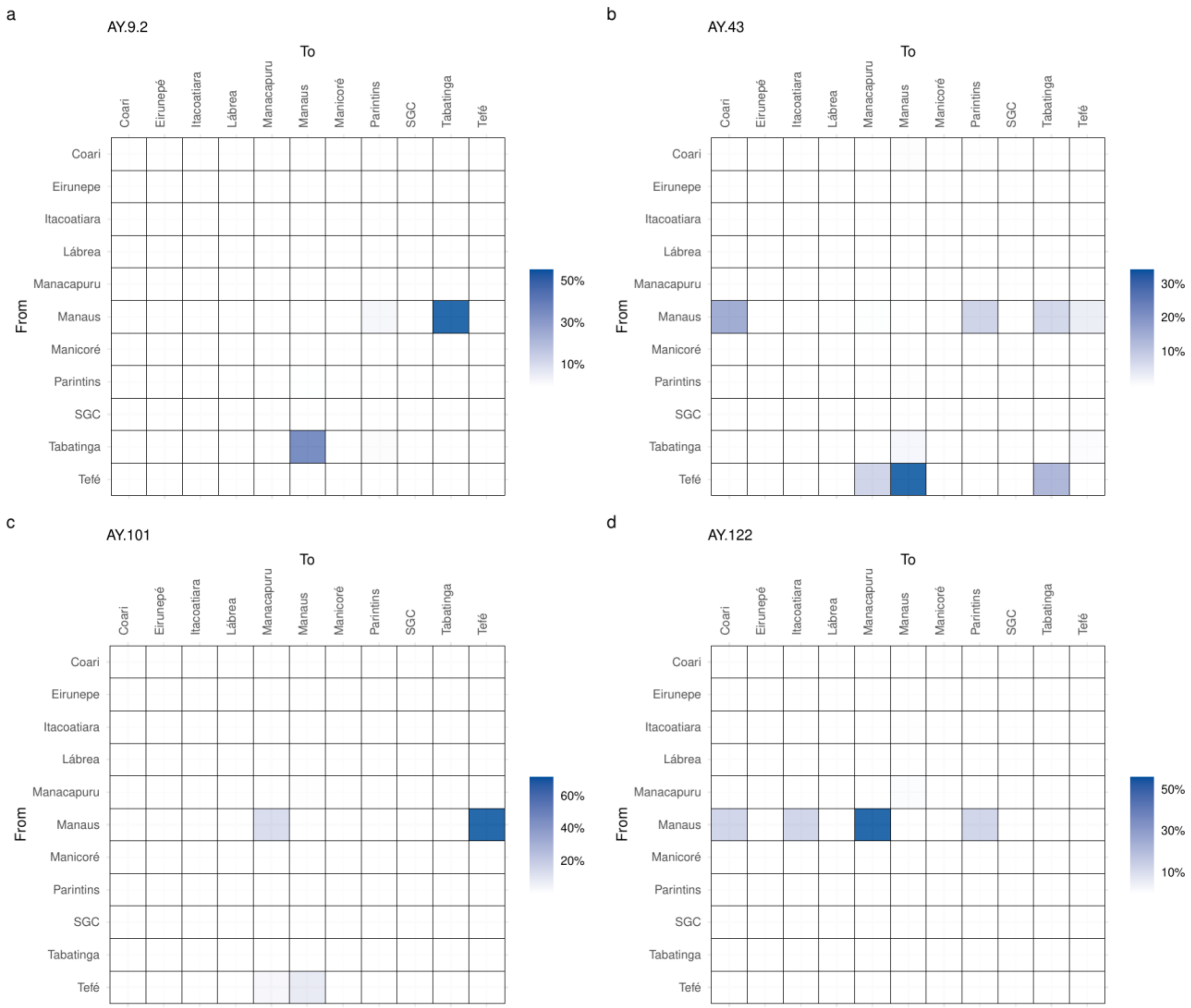

Figure S8: Dissemination of the most prevalent Delta lineages within the Amazonas state. (A-D) Heatmap cells are colored according to the estimated number of migrations between Amazonian regions for Delta's most prevalent lineages. SGC: São Gabriel da Cachoeira.

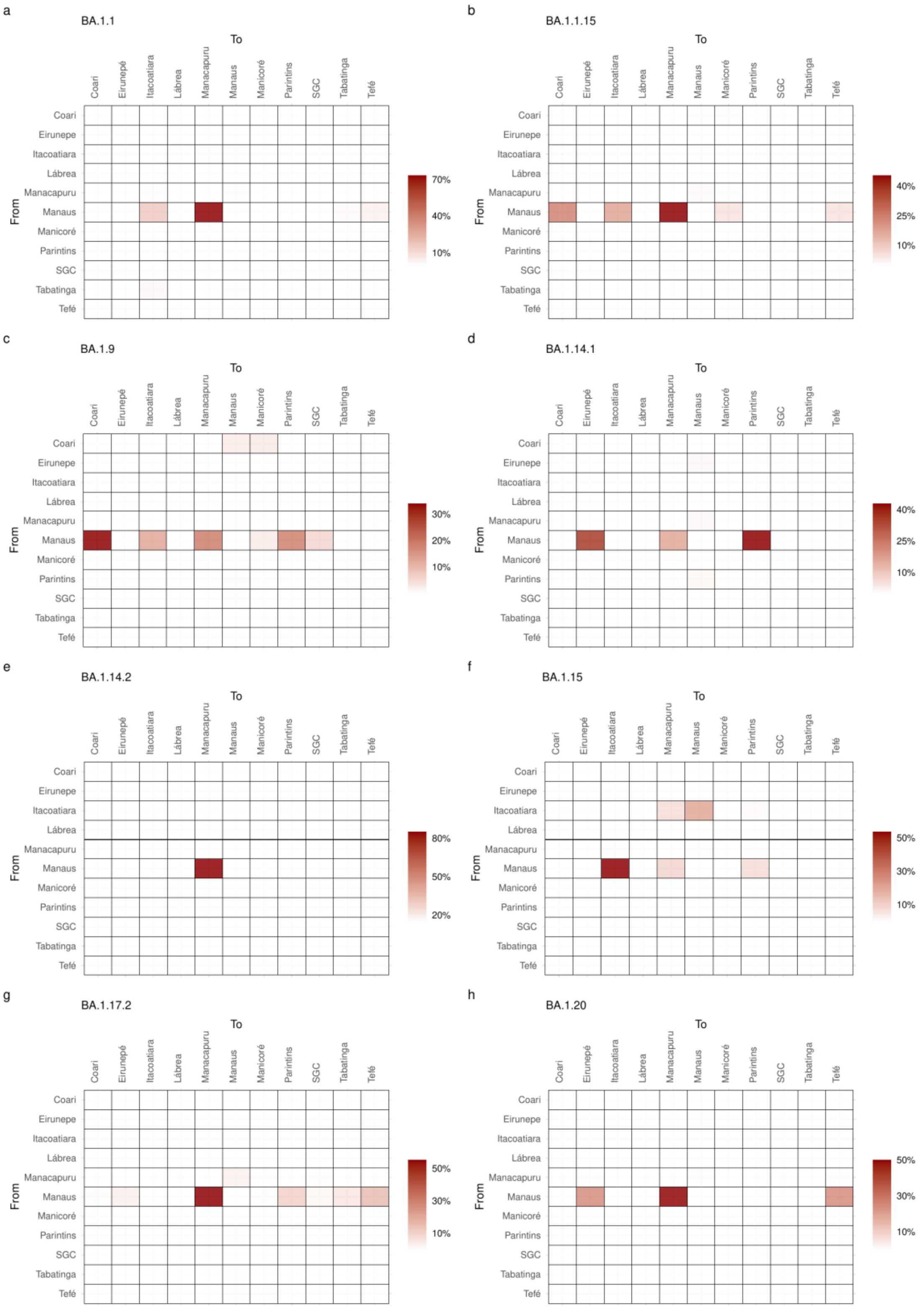

Figure S9: Dissemination of the most prevalent Omicron lineages within the Amazonas state. (A-H) Heatmap cells are colored according to the estimated number of migrations between Amazonian regions for Omicron's most prevalent lineages. SGC: São Gabriel da Cachoeira.

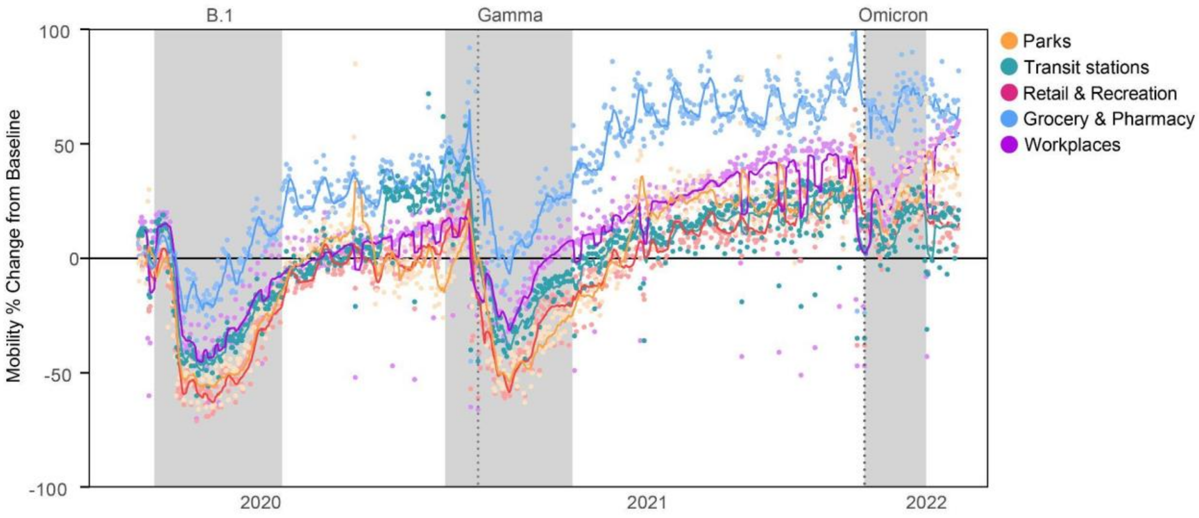

Figure S10: Amazonas daily mobility data between 15th January 2020 and 31st March 2022. Daily mobility data trends are reported as percentage change measured against the baseline. Each place category was colored following the legend in the upper right corner. The three main epidemic waves observed in Manaus: lineage B.1 (March to June 2020), Gamma (December 2020 to March 2021) Omicron (January to February 2022) are highlighted in gray-shaded areas. Vertical dashed lines separate the years.

SUPPLEMENTAL TABLE

Data Availability

GISAID Identifier:	EPI_SET_220913va
doi:	10.55876/gis8.220913va

All genome sequences and associated metadata in this dataset are published in GISAID's EpiCoV database. To view the contributors of each individual sequence with details such as accession number, Virus name, Collection date, Originating Lab and Submitting Lab and the list of Authors, visit 10.55876/gis8.220913va

Data Snapshot

- EPI_SET_220913va is composed of 13,776 individual genome sequences.
- The collection dates range from 2021-01-08 to 2022-01-31;
- Data were collected in 84 countries and territories;
- All sequences in this dataset are compared relative to hCoV-19/Wuhan/WIV04/2019 (WIV04), the official reference sequence employed by GISAID (EPI_ISL_402124). Learn more at https://gisaid.org/WIV04.
